# Fire in the Quill

**DOI:** 10.1371/journal.pbio.1000323

**Published:** 2010-03-09

**Authors:** Armin Schneider

**Affiliations:** Department of Molecular Neurology, Sygnis Bioscience, Heidelberg, Germany

## Abstract

How has our understanding of the brain evolved? And what can its progress tell us about the way science works? Armin Schneider explores these questions in his review of Charles Gross' new collection of essays on the history of neuroscience in *Fire in the Quill*.

**Figure:**
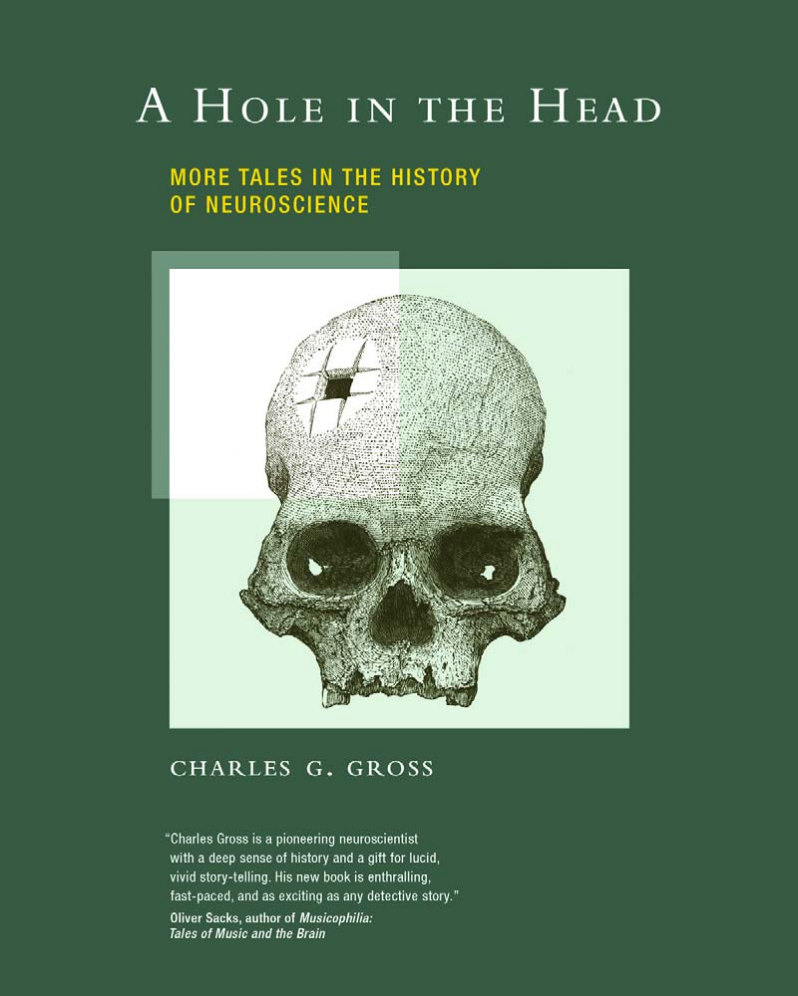
Gross, CG (2009) A hole in the head: more tales in the history of neuroscience. Boston, MA: MIT Press. 366p. ISBN (cloth): 978-0-262-01388-3 US$35.00.

The interest that the discipline “history of neuroscience” receives today among students and teachers of the neurosciences can be witnessed during the annual Society for Neuroscience meeting, where the rows reserved for posters on the topic are fully deserted, and serve as space for a quiet lunch rather than an information and discussion forum. This disinterest is of course partially forced by the need to suck in as much technical detail knowledge as possible in one's field of interest, which in the last 50 years has become narrower and narrower.

But, it may also stem from an underestimation of the importance the non-technical dimensions of the neurosciences have reached today. Theoretical philosophy has increasingly ceased to produce new influences for our thinking, and the sciences—foremost astronomy, physics, and neuroscience—have become a main force that shapes the way we think about ourselves and the world. However, as they venture deeper and deeper into the complex details of their field, scientists are increasingly at a loss to explain the broader implications of what they do to the public.

A prerequisite for the ability to transpose new findings from the level of pure technical understanding to an understanding of its metaphysical and sociological dimensions is to understand how discoveries of the past have shaped the way we perceive the world today.

Thus, a book that helps us to take a few steps back and view neuroscience through a wide-angle lens instead of the microscope objective, while doing so in the most entertaining way, is very welcome indeed.

Charles G. Gross, Professor of Psychology at Princeton University, has contributed seminal work to our understanding of the functioning of the visual cortex and of computation of sensory information in general, and has recently also worked on neurogenesis. His interest in the history of neuroscience dates back to 1960, to his Ph.D. thesis on frontal cortex lesions in the monkey, when he stumbled across Galen's experiments in piglets and got interested in theories on the cortex through the millennia. He has published a considerable number of papers on different topics concerned with the history of neuroscience and is the author of *Vision, Brain, Memory: Tales in the History of Neuroscience*.

His second book, *A Hole in the Head: More Tales in the History of Neuroscience*, is a collection of 12 essays, most of which were written by Gross between 1998 and 2008. The 12 essays are grouped into three main sections: “Early Neuroscience and Its Reverberations Today”; “Neuroscience and Art”; and “Scientists Who Were Before Their Time.” Many essays are left in their original state, but often with a new introduction and annotated by a postscript adding latest developments in the respective field. Gross has clearly broadened the span of topics covered in his previous book, which mainly focused on neuroscience history in the field of vision and cortical function. While this is still a prominent theme, he expands his scope to topics like general physiology, renaissance paintings of anatomy demonstrations, or navigation by ultrasound in bats.

What is the audience the book is intended for? People dealing with the nervous system on a daily basis, such as neuroscientists and neurologists, are the natural target, as well as specialists in medicine and science history. However, the book will be very appealing to interested laymen, who will likely appreciate how Gross blends science history into philosophy and art. Likewise attractive is the lively and entertaining way he restages the historical players and events. The aim for a broader audience is certainly set with the catchy title, and with the front cover praise by Oliver Sacks, the undisputed master of popularizing (in the best sense) the fascinating world of neuropsychology and the workings of the human brain.

Is this aim met? One has to state that the different essays are quite heterogeneous in their depth and accessibility. While the essays in the first two sections are easy to follow without specialized knowledge, the essays in the third section (“Scientists Who Were Before Their Time”) are certainly more difficult to understand for non-scientists. I feel that a few small amendments could have strongly improved the accessibility of the book for the lay readership. First, the introduction to each essay could have summarized in a few sentences the current state of physiological understanding. Second, technical terms could have been explained at their first occurrence (e.g., “phosphene”—a visual sensation often in the form of light flashes that can be artificially induced, for example, by magnetic stimulation of the visual cortex, in the essay “The Fire That Comes from the Eye”).

The book starts almost cautiously with an essay on trepanation—drilling holes in the skull—that is a fair recollection of the history of that procedure done in different cultures and for different reasons. It is in the following essays where Gross really takes up speed and unfolds a firework of wit, humour, and attention to detail when following the fate of ideas through the centuries and millennia. Did you ever wonder why we unnoticingly accept the heart as the seat of love and emotion in hundreds of contemporary pop songs, while consciously knowing that “groove is in the brain”? “Heart versus Brain” is a brilliant essay on the century-long controversy on the location of the mind, interwoven with a vivid biography of Galen, the last rational physician for a long time to come who opposed the prevalent Aristotelian view of the heart as central control organ with his experiment of severing the recurrent laryngeal nerve in a conscious pig. It is with wonder that we follow the perfect logic of Aristotle that relentlessly leads him to the wrong conclusion. But, as in the following essays, it is really the human touch with which Gross depicts his historical cast that I most admired. We come to understand Galen as an ingenious physician with a conceited personality who cleverly exploits public demonstrations to eliminate his opponents and build his career.

“The Fire That Comes from the Eye” explores the historical debate about whether something leaves (extramission) or enters (intromission) the eye when seeing. Although this debate might appear strange with today's knowledge, the extramission view seems to reflect an aspect of basic concept building of our brain, as this is, astonishingly, still the preferred belief among US schoolchildren today.

In “The Discovery of the Motor Cortex” Gross revisits one of his favourite topics and describes the more recent history of cortex physiology. Again, the livelihood of Gross' biographical portraits is stunning: We follow with wonder (and maybe unpleasant memories of our own) the struggle of Ferrier with reviewers for paper acceptance and his problems with emerging animal rights activists. For me, the biggest conceptual achievement in this essay is a novel view on the theories of Gall and Spurzheim, later known as phrenology. While we commonly know phrenology as a dead alley that had a bizarre and unholy renaissance in the Third Reich, Gross convincingly argues that Gall and Spurzheim really paved the way towards a modern understanding of the cortex.

In the section “Neuroscience and Art” Gross subsumes three works on the topic of trepanation in renaissance paintings, left and right in neuroscience and art, and the historical background of Rembrandt's paintings of anatomy demonstrations. While the first and last essays are both highly entertaining and full of precious details, it is the second essay I found highly original. Here, Gross seamlessly amalgamates neuroscience, art, psychology, and philosophy to discuss a problem we do not normally perceive as one—the distinction between left and right. Since Ernst Mach, the great 19th century physicist and psychologist first noted that children constantly confuse b and d or p and q it has become obvious that the distinction between mirror images in a world of bilateral symmetry is a problem that animals and children cannot easily solve.

The last section “Neuroscientists Before Their Time” is a collection of essays on various scientists whose ideas were too novel in their temporal context (or whose peers were too conservative to seriously deal with those). Two excellent pieces in here are on Claude Bernard, the famous French physiologist, and the constancy of the “milieu interieur”; and Donald Griffin's discovery of echolocation in bats. The last paper in the book deals with the question of whether single neurons exist that encode complex features—“grandmother cells”—a topic the author has contributed considerably to with the discovery of hand- and face-selective neurons in the monkey. One piece I feel transgresses the scope of the book is the full reproduction of the translation of Panizza's article on the optic nerve, which is really only something for devoted neuroanatomy history aficionados.

However, the most impressive essay in this section, and long overdue, is on the fate of one Robert Altman, who fled post-war communism in Hungary and, after stays in Germany and Australia, finally came to the United States in 1955, where he stumbled across the discovery of neurogenesis (the fact that new neurons are born daily in the adult brain) during his time at MIT in the 1960s. This discovery can be easily classified as one of the most important conceptual additions to neuroscience in the last 30 years and revoked the long standing dogma from Cajal's times that after development no new neurons are added to the brain. However, not only were his revolutionary findings ignored, but he was denied tenure at MIT, faced frequent rejections of his subsequent papers, and eventually lost all grant support. Finally, he resolved to finance his lab at Purdue with the publication of brain atlases. Why was this discovery ignored for almost 30 years? Gross unveils with great clarity that the reasons for this are deeply rooted in the way science works, where a peer culture dominated by prominent scientists determines grant support and publications, a process which ultimately favours perpetuation of tradition over revolutionary ideas. Two factors contributing to Altman's case were that he was not educated in one of the prominent labs in the field and influential scientists failed to reproduce his findings.

Culminated in Altman's story is one of the most valuable lessons that scientists and non-scientists alike can learn from the book; our current beliefs and truths, like the beliefs of the people before us, are transient in nature and subject to the challenge of time. We must constantly remind ourselves to remain open to the future.

